# MiR-218 Impairs Tumor Growth and Increases Chemo-Sensitivity to Cisplatin in Cervical Cancer

**DOI:** 10.3390/ijms131216053

**Published:** 2012-11-28

**Authors:** Jiarui Li, Zhang Ping, Hui Ning

**Affiliations:** 1 Department of Gynecology, Xin Hua Hospital Affiliated to Shanghai Jiao Tong University School of Medicine, 1665 Kongjiang Road, Shanghai 200092, China; E-Mails: jiaruili_nj@yeah.net (J.L.); xiangming_nj@163.com (Z.P.); 2 Department of Gynecology and Obstetrics, Changhai Hospital, Second Military Medical University, 168 Changhai Road, Shanghai 200433, China

**Keywords:** miR-218, cervical cancer, HeLa, cisplatin

## Abstract

MicroRNAs are noncoding RNA molecules of 18–25 nucleotides that regulate gene expression at the post-transcriptional levels. Recent data revealed that miR-218 played key roles in tumor metastasis. Here, we described the regulation and function of miR-218 in cervical cancer. Overexpression of miR-218 reduced the proliferation of the human cervical cancer cell line HeLa and induced cell apoptosis through the AKT-mTOR signaling pathway. In addition, it forced expression of miR-218 suppressed tumor growth in the orthotopic mouse model of HeLa cells. Furthermore, miR-218 increased chemosensitivity to cisplatin (CDDP) *in vitro*. Our results indicated that targeting miR-218 may provide a strategy for blocking the development of cervical cancer.

## 1. Introduction

Cervical cancer (CC), as the second leading cause of cancer morbidity and mortality for women around the world, is the term for a malignant neoplasm arising from cells originating in the cervix uteri. One of the most common symptoms of cervical cancer is abnormal vaginal bleeding, but in some cases there may be no obvious symptoms until the cancer has progressed to an advanced stage [1]. Clinical drugs did not show strong therapeutic efficacy combined with a lack of side effects. According to the American Cancer Society, there are no established guidelines to prevent cervical cancer. To reduce the high disease burden, the development of effective prevention and therapeutic procedures is needed.

It is well-known that infection with the high risk human papilloma virus (HPV) is the predominant risk factor for CC [2]. Since most infected females do not develop the disease, other factors must contribute to the initiation of the cancer. Recent studies have shown that aberrant activation of mTOR is involved in many cancers and the activation status of the mTOR pathway in cervical cancer has been investigated [3]. In the present study, the high expression of the mTOR pathway in pathological cervical tissues was observed, as well as in cervical cancer cell line. Other cancer models have also identified mTOR signaling as a potential target for anticancer therapy [4–6].

MicroRNAs (miRNAs) are small, conserved, non-coding short RNAs of 18–25 nucleotides in length that bind to target mRNAs mainly at their 3′-UTR [7]. Many microRNAs have been implicated as key regulators of cellular growth and differentiation and have been found to dysregulate proliferation in human tumors [8–10]. Cancer-linked microRNAs also alter the epigenetic landscape by way of DNA methylation and post-translational modifications of histones [11].

In human cancers, the expression of miRNAs is generally downregulated in malignant tissues compared with the corresponding nonmalignant tissues, suggesting the deregulation of miRNA expression and the contribution of miRNAs to the multistep processes of carcinogenesis, either as oncogenes or as tumor-suppressor genes [12–14]. In our studies, overexpression of miR-218 could inhibit the growth of human cervical cancer cell line HeLa both *in vivo* and *in vitro*. Furthermore, the treatment with miR-218 promoted chemosensitivity to cisplatin (CDDP) of HeLa cells *in vitro*. Hence, targeting miR-218 may provide a novel strategy for the treatment of cervical cancer.

## 2. Results and Discussion

### 2.1. Results

#### 2.1.1. Tumor Suppressive Effect of miR-218 in the Proliferation of Cervical Cancer Cell Growth

**Figure 1:**
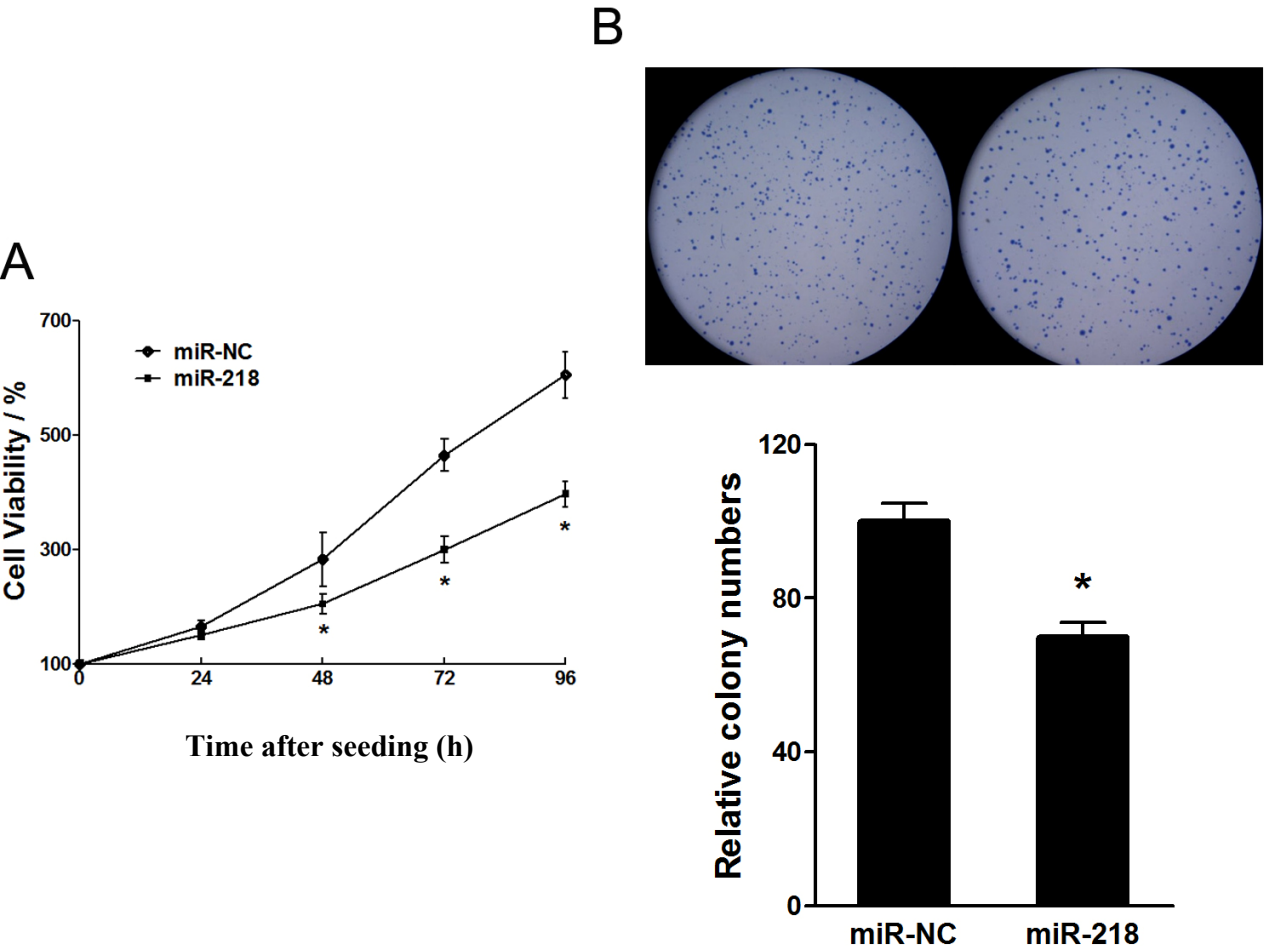
Overexpression of miR-218 reduced proliferation of cervical cancers. (**A**) miR-218 overexpression decreased HeLa cell growth. Cell viability was measured using a WST-1 kit at indicated time point. Data were means ± SD from three independent experiments performed in sextuple. (**B**) miR-218 overexpression reduced colony formation of HeLa cells. Five thousand cells were mixed with resolved agarose and seeded in 6-well plates for three weeks. Colonies were stained by crystal violet (top) and counted (bottom). Data were means ± SE. ^*^ indicated significant differences between groups of miR-218 and control. ^*^p < 0.05.

MiR-218 is commonly downregulated in cervical cancers [15]. To explore the biological roles of miR-218 in the cervical cells, we stably overexpressed miR-218 in HeLa cells (HeLa/miR-218) by transfecting plasimids carrying miR-218 gene and then selected by puromycin. Also, we established HeLa/miR-NC as control. The overexpression of miR-218 in HeLa/miR-218 cells was confirmed by RT-PCR (See Supplement Figure S1). The stable cells were seeded in 96-well plates and measured by WST-1 kit for cell growth at indicated time points. Overexpression of miR-218 markedly inhibited the cell growth rate of HeLa cells (Figure 1A). An anchorage independent colony formation assay indicated that miR-218 reduced the numbers of colonies of HeLa cells (Figure 1B), which further demonstrated the inhibition activities of miR-218 in cervical cancer cell proliferation.

#### 2.1.2. Overexpression of miR-218 Inhibited Expression of Rictor, an mTOR Component, and Its Downstream Pathway

**Figure 2:**
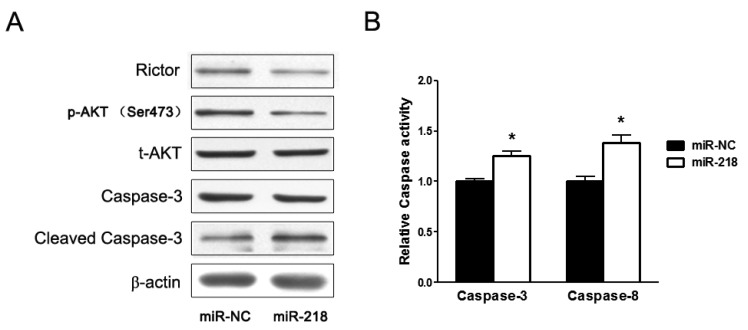
MiR-218 inhibited expression of Rictor, a component of mTOR, and induced apoptosis of cervical cancer cells. (**A**) Western blotting assay of miR-218 overexpression cells. Total cell lysats were subjected to analysis the levels of Rictor, p-AKT, total-AKT, caspase-3 and cleaved caspase-3. (**B**) Overexpression of miR-218 increased activities of caspase. Cells were transfected with 40 nM of pre-miR-218 or pre-miR-NC for 72 h and subjected to caspase enzyme activity analysis. Data were means ± SE from three experiments. ^*^ represented significant differences between groups of miR-218 and control. ^*^p < 0.05.

Rictor (rapamycin-insensitive companion of mTOR) is direct target of miR-218 which was validated in oral cancer cells before [13]. Here in cervical cancer HeLa cells, we demonstrated that miR-218 also downregulated the protein levels of Rictor (Figure 2A). Rictor, together with mTOR, forms mTOR complex 2 (mTORC2), directly regulates the phosphorylation of AKT at Ser-473. In this study, we found that levels of phospho-AKT (Ser-473) were reduced when miR-218 was overexpressed, while the total protein levels of AKT were not obviously changed. In addition, ectopic expression of miR-218 significantly increased protein levels of cleaved Caspase-3, a symbol of cell apoptosis. Furthermore, the activity analysis indicated that ectopic expression of miR-218 significantly activated both Caspase-3 and -8 (Figure 2B).

#### 2.1.3. MiR-218 Impaired *In Vivo* Tumor Growth

**Figure 3:**
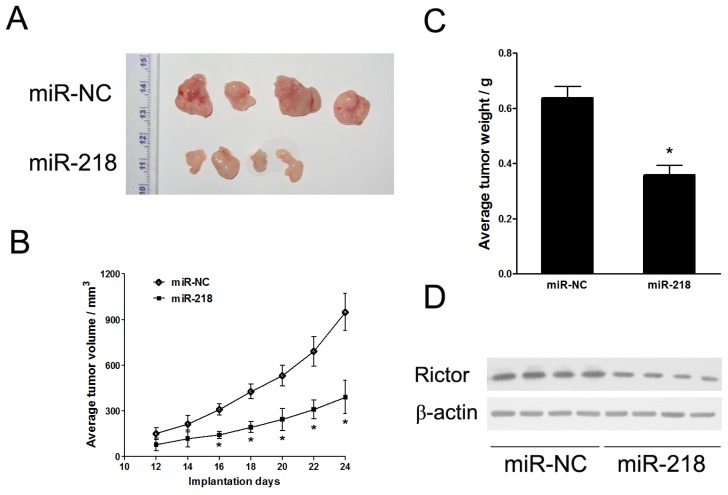
Forced expression of miR-218 impaired cervical tumor growth *in vivo*. Cells stably expressing miR-218 or miR-NC were suspended in FBS-free DMEM medium and subcutaneously injected into 2 sides of posterior flanks of nude mice (*n* = 4). 24 days after implantation, mice were sacrificed and xenografts were removed. (**A**) Representative tumor xenografts at day 24. (**B**) Tumor volumes were measured every two days from the time that they were apparent. (**C**) Average weights of tumors. (**D**) Western blotting analysis of Rictor protein expression in xenograft tumors. Data were means ± SE. ^*^ indicated significant differences between groups of miR-218 and control. ^*^p < 0.05.

To further explore the roles of miR-218 in *in vivo* tumor growth, we employed ectopic transplantation model in nude mice. Stable cell lines, HeLa/miR-NC and HeLa/miR-218, were subcutaneously injected into both posterior flanks of nude mice, respectively. Tumors were monitored every two days from the time that they were apparent. Compared with control group, tumor growth of miR-218 group was significantly reduced (Figure 3A,B). 24 days after implantation, mice were sacrificed. The tumor xenografts were removed out and weighed (Figure 3C). Consistent with tumor volumes, the xenograft weights were decreased by miR-218 expression. Western blotting analysis revealed that protein levels of Rictor were aberrantly inhibited in the miR-218 overexpression group (Figure 3D). These data indicated that miR-218 acted as a tumor suppressor in cervical cancer.

#### 2.1.4. MiR-218 Increased Chemosensitivity of Cervical Cancer Cells to Cisplatin via Its Target Rictor

**Figure 4:**
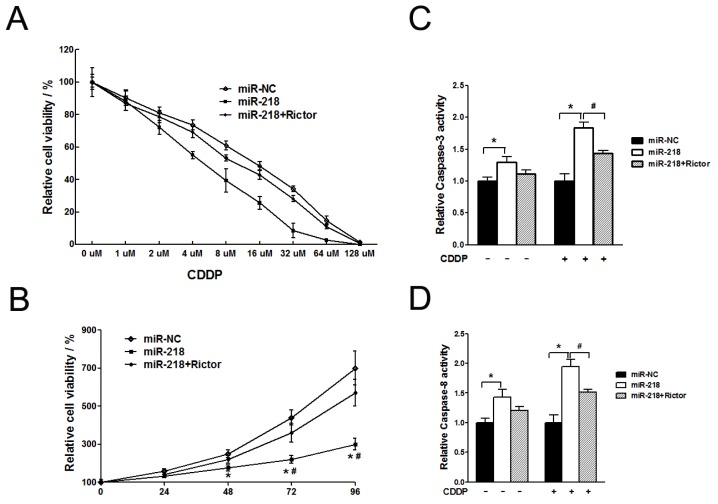
MiR-218 increased chemosensitivity to cisplatin (CDDP), which was counteracted by overexpression of Rictor. Cells were transfected with 40nM pre-miR-NC, or 40nM pre-miR-218 with or without Rictor cDNA. Eight hours after transfection, cells were exposed to CDDP for further examination. (**A**) Proliferation assay of tumor cells in the present of different concentration of CDDP. 5000 cell per well were seeded in 96-well plates and incubated with different concentration of CDDP for 72 h. Cell viability was measured by WST-1 kit. Data were means ± SE from three independent experiments performed in sextuple. (**B**) Cell proliferation in the treatment of 12 μM CDDP. Data were means ± SE from three independent experiments performed in sextuple. (**C**, **D**) Caspase activity analysis of tumor cells with or without CDDP. Cells treated with or without 12 μM CDDP were analyzed for Caspase-3 (**C**) and Capase-8 (**D**), respectively. ^*^ represented as significant differences between groups of miR-218 and control, while ^#^ represented as significant differences between groups of miR-218 and miR-218+Rictor. ^*,#^*p* < 0.05.

We constructed adenovirus carrying Rictor (Ad-Rictor) to rescue the low protein levels of Rictor in HeLa/miR-218 cells. To investigate whether miR-218 and its target, Rictor, play roles in the chemotherapy of cervical cancer, we exposed the stable cell lines, HeLa/miR-NC, HeLa/miR-218 or HeLa/miR-218 infected with Ad-Rictor, with different concentration of cisplatin (CDDP) ranging from 0 to 128 μM for 72h (Figure 4A). The cell viability was measured using WST-1 method. Overexpression of miR-218 increased sensitivity of HeLa cells to CDDP, while restoration of Rictor reversed it and increased chemo-resistance to that of HeLa/miR-NC cells. As shown in Table 1, the IC50 of the three groups were 15.85 ± 1.21, 5.96 ± 0.57 and 11.88 ± 0.94, respectively, which indicated that miR-218 significantly increased chemosensitivity to CDDP. To investigate the role of miR-218 in CDDP treated cells, we detected the proliferation and apoptosis effect of miR-218-overexpressing cells exposed in CDDP. The three groups of cells were treated with 10 uM of CDDP (~2 × IC50 of HeLa/miR-218 stable cells) for 72 h. WST-1 assay showed a remarkably decrease of cell proliferation (Figure 4B) and activities of Caspase-3 and -8 (Figure 4C,D) of miR-218 overexpressing cells. Furthermore, Rictor protein levels were reduced in the miR-218 transfectant and its levels were restored in the miR-218-Rictor co-transfectant (See Supplement Figure S2).

### 2.2. Discussion

The key finding of our study is that the tumor suppressive microRNA miR-218 reduced the growth of tumor cells and inhibited AKT-mTOR signaling pathway in cervical cancer. In addition, miR-218 increased chemosensitivity to cisplatin (CDDP) *in vitro* via its target, Rictor, which was evaluated as cell apoptosis as a measure of chemosensitivity.

The mTOR signaling molecules are activated in cervical cancer cell lines and cervical tumors. Activation of the mTOR signaling pathway may contribute to tumor cell proliferation and survival of cervical cancer cells [16,17], and has been recognized as a key therapeutic target for the treatment of several types of cancers [18]. Rictor is a critical component of mTORC2 that is required for assembly and activity of the PDK-2 kinase [19]. Therefore, lower Rictor levels would be expected to reduce the phosphorylation of AKT (Ser 473). As shown in Figure 2, miR-218 inhibited the level of both Rictor and Phospho-AKT in HeLa cells. And recent study has shown that miR-218 targeted the mTOR component Rictor and inhibited AKT phosphorylation in oral cancer [13]. Therefore, it suggested that miR-218 suppressed the growth of tumor originated from HeLa cells by AKT-mTOR signaling pathway.

Cisplatin, one of the broadest-spectrum anticancer agents, is currently used in the treatment of many types of advanced cancer, including carcinoma of the cervix [20]. Until now, the prognosis of patients with advanced, persistent, or recurrent squamous cell carcinoma of the cervix has been poor [21,22]. Currently, the most effective systemic treatment for metastatic cervical cancer consists of cisplatin-based combination chemotherapy [23]. Unfortunately, although CC has been shown to be cisplatin-sensitive, responses are not typically durable, and the majority of patients experience subsequent disease progression [24]. Resistance to chemotherapy is the most frequent obstacle to effective treatment [25,26]. Pre-treatment with rapamycin inhibited activation of mTOR signaling and significantly enhanced the sensitivity of HeLa cells to cisplatin, by increasing apoptotic cell death [27,28]. Furthermore, it is possible that miR-218 enhanced chemosensitivity to cisplatin by the AKT-mTOR signaling pathway. The tumor suppressive microRNA miR-218 also targeted the mTOR component Rictor and blocked AKT phosphorylation in oral cancer [13], inhibited invasion and metastasis of gastric cancer by targeting the Robo1 receptor [29].

MicroRNAs (miRNAs), one such class of non-coding RNAs, have been implicated in the regulation of cell growth, differentiation, and apoptosis [30–32]. While their study is still at an early stage and their mechanism of action along with their importance in cancer is not yet fully understood, they may provide an important layer of genetic regulation in tumorigenesis, and ultimately become valuable therapeutic tools.

## 3. Experimental Section

### 3.1. Cell Culture

A human cervical cancer cell line HeLa was cultured in DMEM medium supplemented with 10% FBS and 1% penicillin/streptomycin and maintained at 37 °C in a humidified incubator containing 5% CO2.

### 3.2. Generation of HeLa Cells with Stable Expression of miR-218

HeLa cells were selected and maintained in DMEM complete medium containing 1 mg/mL G418 (Invitrogen) after transfection with pCR-miR-218 or pCR-miR-NC (control). After 2–4 weeks selection, the remaining cells were stably overexpressed with miR-218 or miR-NC.

### 3.3. Cell Proliferation Assay

Cells were seeded at 1000 per well in 96-well plates. Cell viability was evaluated using a WST-1 kit (Roche) according to the manufacturer’s instruction at indicated time. All results were from three separate experiments with six replicates.

### 3.4. Anchorage-Independent Colony Formation Assay

For anchorage-independent colony formation assay, 1 mL of 1% agarose (Sigma, Saint Louis, MI, USA) was added to each well of 6-well plates and kept in 4 °C, and pre-warm in 37 °C before use. Five thousand cells were mixed with 0.5 mL of 0.5% agarose and added onto the top of the well. About 2–3 weeks later, colonies were fixed with methanol for 15 min and stained with 0.1% crystal violet. Colonies with diameter more than 1.5 mm were counted. Experiments were performed with three replicates for three times.

### 3.5. Western Blotting Assay

Cells were harvested and lysed on ice for 30 min inRIPA buffer (Beyotime) supplemented with 1 mM phenylmethylsulfonyl fluoride (PMSF). Lysates were centrifuged at 12,000 rpm for 15 min, and supernatants were collected. Total proteins were separated by SDS-PAGE, and transferred onto nitrocellulose membranes in transfer buffer (20 mM Tris, 150 mM glycine, 20% (*v*/*v*) methanol). Membranes were blocked with 5% nonfat dry milk in PBS containing 0.05% Tween-20, and incubated with antibodies against Rictor, phospho-AKT (Ser-473), total AKT, Caspase-3, cleaved Caspase-3 (Cell Signaling Technology, Danvers, MA, USA) or β-actin (Sigma).

### 3.6. Caspase Activity

The caspase activity evaluation was described as before [33]. In brief, cytosolic protein extracts were harvested and homogenized in isolation buffer containing 10 mM Tris-HCl buffer (pH 7.6), 5 mM MgCl_2_, 1.5 mM potassium acetate, 2 mM dithiothreitol and protease inhibitor mixture tablets (Roche Applied Science, Mannheim, Germany). General caspase-3 and -8 activities were determined by enzymatic cleavage of chromophore p-nitroanilide (pNA) from the substrates N-acetyl-Asp-Glu-Val-Asp-pNA (DEVD-pNA) and N-acetyl-Ile-Glu-pro-Asp-pNA (IEPD-pNA) (Sigma), respectively. The proteolytic reaction was carried out in isolation buffer containing 50 μg of cytosolic protein and 50 μM specific caspase substrate. The reaction mixtures were incubated at 37 °C for 1 h, and measured by monitoring A405 using a 96-well plate reader.

### 3.7. *In Vivo* Tumorigenesis Study

Female 6–8 weeks BALB/c nude mice were from Shanghai Laboratory Animal Center (Chinese Academy of Sciences, Shanghai, China) and maintained in special pathogen-free (SPF) condition. Animal experimental procedures were approved by the Animal Ethics Committee of Nanjing Medical University. 4 × 10^6^ of cells were suspended in FBS-free DMEM medium and subcutaneously injected into two sides of the posterior flanks of nude mice. Tumor sizes were monitored every two days at day 10 when they were apparently seen. Tumor volume was calculated as follows: volume = 0.5 × Length × Width^2^. 24 days after implantation, mice were sacrificed and tumors were removed.

### 3.8. Adenovirus Preparation

Recombinant adenoviruses were constructed using the AdEasy system. The construction of adenovirus carrying Rictor was performed as described before [34]. Briefly, Rictor cDNA fragment was PCR amplified using pDONR-Rictor (GeneCopoeia) as template, sequenced and inserted into into pAdtrack-CMV shuttle vector. The resulting plasmid pAdTrack-CMV-Rictor was linearized with *Pme*I followed by homologous recombination with bone plasmid pAdEasy-1 in BJ5183 bacteria cells to generate recombinant plasmid pAd-Rictor, which was then digested with *Pac*I and transfected to AD-293 cells by Lipofectamine 2000 (Invitrogen, Carlsbad, CA, USA) to package recombinant adenovirus. The primers used were as follows: Rictor-F (*Sal*I), 5′-GTCGACATGGCGGCGAT CGGCCGCGGCC-3′; Rictor-R (EcoR V), 5′-GATATCTCAGGATTCAGCAGATGTATC-3′. Viral titers were determined using the BD Adeno-X Rapid Titer kit (BD Biosciences, Franklin Lakes, NJ, USA) according to the manufacturer’s manual.

### 3.9. *In Vitro* Assay of Chemosensitivity to Cisplatin (CDDP)

Cell were seeded at density of 4000 cells/well in 96-well plates and allowed to attach overnight. CDDP (Sigma) was freshly prepared and added to cells with final concentration series from 0 to 128 μM. After incubation for 72 h, cell viability was determined by WST-1 kit. The IC50 values were calculated and presented as means ± SE from three independent experiments performed in sextuple.

### 3.10. CDDP Treatment of Cancer Cell *in Vitro*

Cells were seeded and allowed to attach overnight. On the second day, freshly prepared CDDP was added. The concentration of CDDP was 2 × IC50 of HeLa/miR-218 stable cells. The chemotherapeutical-treated cells were further cultured for cell proliferation assay at indicated time points or for caspase activity assay for 72 h.

### 3.11. Statistical Analysis

Data in the present study were represented as means ± SD of at least three independent experiments except as indicated. Student’s unpaired *t* test was used for comparison between two groups. Values were considered significantly different when *p* < 0.05.

## 4. Conclusions

We have shown that miR-218 inhibited the proliferation of the human cervical cancer cell line HeLa and increased chemosensitivity to cisplatin *in vitro* by blocking the AKT-mTOR signaling pathway, which may provide an important layer of genetic regulation in tumorigenesis and ultimately become valuable therapeutic tools.

## Supplementary Information



## Figures and Tables

**Table 1:** IC_50_ (72 h) of CDDP treated cancer cells.

Stable Cell Line	IC50 (μM) of Cells Treated by CDDP
HeLa/miR-NC	15.85 ± 1.21
HeLa/miR-218	5.96 ± 0.57 *,#
HeLa/miR-218 + Rictor	11.88 ± 0.94

* indicated significant differences between groups of miR-218 and control.

# represented significant differences between groups of miR-218 and miR-218+Rictor.

*,# *p* < 0.05.

## References

[1] Shi J.F., Chen J.F., Canfell K., Feng X.X., Ma J.F., Zhang Y.Z., Zhao F.H., Li R., Ma L., Li Z.F. (2012). Estimation of the costs of cervical cancer screening, diagnosis and treatment in rural Shanxi Province, China: A micro-costing study. BMC Health Serv. Res.

[2] Lea J.S., Sunaga N., Sato M., Kalahasti G., Miller D.S., Minna J.D., Muller C.Y. (2007). Silencing of HPV 18 oncoproteins With RNA interference causes growth inhibition of cervical cancer cells. Reprod. Sci.

[3] Ji J., Zheng P.S. (2010). Activation of mTOR signaling pathway contributes to survival of cervical cancer cells. Gynecol. Oncol.

[4] Noh W.C., Kim Y.H., Kim M.S., Koh J.S., Kim H.A., Moon N.M., Paik N.S. (2008). Activation of the mTOR signaling pathway in breast cancer and its correlation with the clinicopathologic variables. Breast Cancer Res. Treat.

[5] Marinov M., Ziogas A., Pardo O.E., Tan L.T., Dhillon T., Mauri F.A., Lane H.A., Lemoine N.R., Zangemeister-Wittke U., Seckl M.J. (2009). AKT/mTOR pathway activation and BCL-2 family proteins modulate the sensitivity of human small cell lung cancer cells to RAD001. Clin. Cancer Res.

[6] Babcock J.T., Quilliam L.A. (2011). Rheb/mTOR activation and regulation in cancer: Novel treatment strategies beyond rapamycin. Curr. Drug Targets.

[7] Yanokura M., Banno K., Kobayashi Y., Kisu I., Ueki A., Ono A., Masuda K., Nomura H., Hirasawa A., Susumu N. (2010). MicroRNA and endometrial cancer: Roles of small RNAs in human tumors and clinical applications (Review). Oncol. Lett.

[8] Jiang Y.W., Chen L.A. (2012). microRNAs as tumor inhibitors, oncogenes, biomarkers for drug efficacy and outcome predictors in lung cancer (review). Mol. Med. Report.

[9] Kelly B.D., Miller N., Healy N.A., Walsh K., Kerin M.J. (2012). A review of expression profiling of circulating microRNAs in men with prostate cancer. BJU Int..

[10] Nair V.S., Maeda L.S., Ioannidis J.P. (2012). Clinical outcome prediction by microRNAs in human cancer: A systematic review. J. Natl. Cancer Inst.

[11] Lujambio A., Calin G.A., Villanueva A., Ropero S., Sanchez-Cespedes M., Blanco D., Montuenga L.M., Rossi S., Nicoloso M.S., Faller W.J. (2008). A microRNA DNA methylation signature for human cancer metastasis. Proc. Natl. Acad. Sci. USA.

[12] Lages E., Ipas H., Guttin A., Nesr H., Berger F., Issartel J.P. (2012). MicroRNAs: Molecular features and role in cancer. Front. Biosci.

[13] Uesugi A., Kozaki K., Tsuruta T., Furuta M., Morita K., Imoto I., Omura K., Inazawa J. (2011). The tumor suppressive microRNA miR-218 targets the mTOR component Rictor and inhibits AKT phosphorylation in oral cancer. Cancer Res.

[14] Martinez I., Gardiner A.S., Board K.F., Monzon F.A., Edwards R.P., Khan S.A. (2008). Human papillomavirus type 16 reduces the expression of microRNA-218 in cervical carcinoma cells. Oncogene.

[15] Zhou X., Chen X., Hu L., Han S., Qiang F., Wu Y., Pan L., Shen H., Li Y., Hu Z. (2010). Polymorphisms involved in the miR-218-LAMB3 pathway and susceptibility of cervical cancer, a case-control study in Chinese women. Gynecol. Oncol.

[16] Rostaing L., Kamar N. (2010). mTOR inhibitor/proliferation signal inhibitors: Eentering or leaving the field?. J. Nephrol.

[17] Morgensztern D., McLeod H.L. (2005). PI3K/Akt/mTOR pathway as a target for cancer therapy. Anticancer Drugs.

[18] Guo C., Gasparian A.V., Zhuang Z., Bosykh D.A., Komar A.A., Gudkov A.V., Gurova K.V. (2009). 9-Aminoacridine-based anticancer drugs target the PI3K/AKT/mTOR, NF-kappaB and p53 pathways. Oncogene.

[19] Breuleux M., Klopfenstein M., Stephan C., Doughty C.A., Barys L., Maira S.M., Kwiatkowski D., Lane H.A. (2009). Increased AKT S473 phosphorylation after mTORC1 inhibition is rictor dependent and does not predict tumor cell response to PI3K/mTOR inhibition. Mol. Cancer Ther.

[20] Brewer C.A., Blessing J.A., Nagourney R.A., McMeekin D.S., Lele S., Zweizig S.L. (2006). Cisplatin plus gemcitabine in previously treated squamous cell carcinoma of the cervix: A phase II study of the Gynecologic Oncology Group. Gynecol. Oncol.

[21] Long H.J., Monk B.J., Huang H.Q., Grendys E.C., McMeekin D.S., Sorosky J., Miller D.S., Eaton L.A., Fiorica J.V. (2006). Clinical results and quality of life analysis for the MVAC combination (methotrexate, vinblastine, doxorubicin, and cisplatin) in carcinoma of the uterine cervix: A Gynecologic Oncology Group study. Gynecol. Oncol..

[22] Anders J.C., Grigsby P.W., Singh A.K. (2006). Cisplatin chemotherapy (without erythropoietin) and risk of life-threatening thromboembolic events in carcinoma of the uterine cervix: The tip of the iceberg? A review of the literature. Radiat. Oncol.

[23] Coleman R.E., Clarke J.M., Slevin M.L., Sweetenham J., Williams C.J., Blake P., Calman F., Wiltshaw E., Harper P.G. (1990). A phase II study of ifosfamide and cisplatin chemotherapy for metastatic or relapsed carcinoma of the cervix. Cancer Chemother. Pharmacol.

[24] Nagai Y., Toita T., Wakayama A., Nakamoto T., Ooyama T., Tokura A., Inamine M., Kudaka W., Murayama S., Aoki Y. (2012). Concurrent chemoradiotherapy with paclitaxel and cisplatin for adenocarcinoma of the cervix. Anticancer Res.

[25] Nakada S., Aoki D., Ohie S., Horiuchi M., Suzuki N., Kanasugi M., Susumu N., Udagawa Y., Nozawa S. (2005). Chemosensitivity testing of ovarian cancer using the histoculture drug response assay: Sensitivity to cisplatin and clinical response. Int. J. Gynecol. Cancer.

[26] Calabro A., Singletary S.E., Tucker S., Boddie A., Spitzer G., Cavaliere R. (1989). *In vitro* thermo-chemosensitivity screening of spontaneous human tumors: Significant potentiation for cisplatin but not adriamycin. Int. J. Cancer.

[27] Peng D.J., Wang J., Zhou J.Y., Wu G.S. (2010). Role of the Akt/mTOR survival pathway in cisplatin resistance in ovarian cancer cells. Biochem. Biophys. Res. Commun.

[28] Wangpaichitr M., Wu C., You M., Kuo M.T., Feun L., Lampidis T., Savaraj N. (2008). Inhibition of mTOR restores cisplatin sensitivity through down-regulation of growth and anti-apoptotic proteins. Eur. J. Pharmacol.

[29] Tie J., Pan Y., Zhao L., Wu K., Liu J., Sun S., Guo X., Wang B., Gang Y., Zhang Y. (2010). MiR-218 inhibits invasion and metastasis of gastric cancer by targeting the Robo1 receptor. PLoS Genet.

[30] Boeri M., Pastorino U., Sozzi G. (2012). Role of MicroRNAs in Lung Cancer: MicroRNA Signatures in Cancer Prognosis. Cancer J.

[31] Li X., Wang Q., Zheng Y., Lv S., Ning S., Sun J., Huang T., Zheng Q., Ren H., Xu J. (2011). Prioritizing human cancer microRNAs based on genes’ functional consistency between microRNA and cancer. Nucleic Acids Res.

[32] Brenner B., Hoshen M.B., Purim O., David M.B., Ashkenazi K., Marshak G., Kundel Y., Brenner R., Morgenstern S., Halpern M. (2011). MicroRNAs as a potential prognostic factor in gastric cancer. World J. Gastroenterol.

[33] Borralho P.M., Kren B.T., Castro R.E., da Silva I.B., Steer C.J., Rodrigues C.M. (2009). MicroRNA-143 reduces viability and increases sensitivity to 5-fluorouracil in HCT116 human colorectal cancer cells. FEBS J.

[34] Luo J., Deng Z.L., Luo X., Tang N., Song W.X., Chen J., Sharff K.A., Luu H.H., Haydon R.C., Kinzler K.W. (2007). A protocol for rapid generation of recombinant adenoviruses using the AdEasy system. Nat. Protocols.

