# Supplementary material for: MiR-218 Impairs Tumor Growth and Increases Chemo-Sensitivity to Cisplatin in Cervical Cancer
Source: Int J Mol Sci. 2012 Nov 28;13(12):16053–64. doi: 10.3390/ijms131216053 (PMC3546678; doi:10.3390/ijms131216053)
Supplement: Supplementary file 1 [file ijms-13-16053-s001.pdf]

Supplementary Information

Figure S1. RT-PCR analysis confirming that miR-218 was overexpressing in HeLa/miR-218 cells.

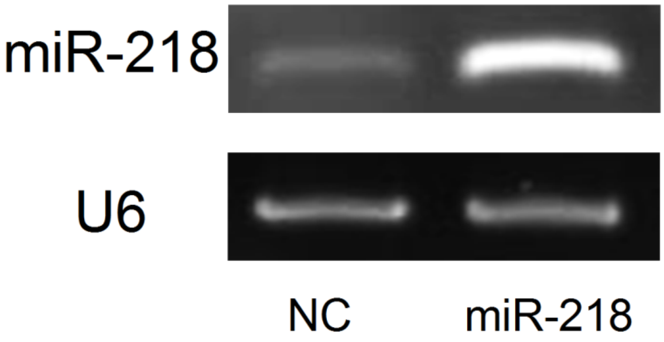

Figure S2. Western bolt analysis of Rictor expression in HeLa/miR-218 cells.

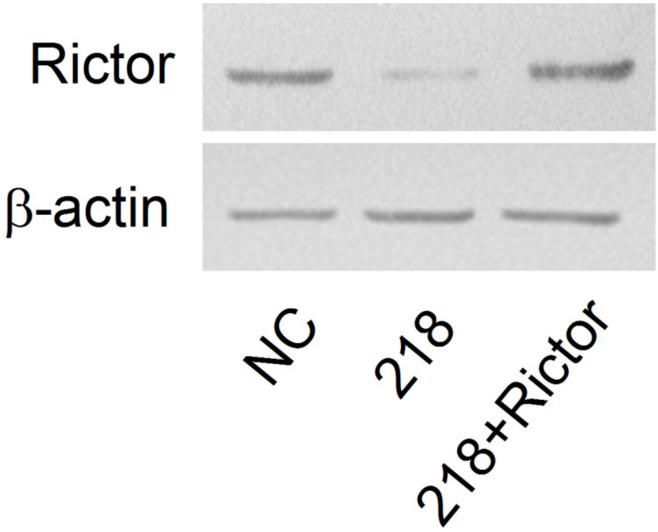

© 2012 by the authors; licensee MDPI, Basel, Switzerland. This article is an open access article distributed under the terms and conditions of the Creative Commons Attribution license (http://creativecommons.org/licenses/by/3.0/).
